# Circular RNA circSLC25A16 contributes to the glycolysis of non-small-cell lung cancer through epigenetic modification

**DOI:** 10.1038/s41419-020-2635-5

**Published:** 2020-06-08

**Authors:** Hong Shangguan, Hong Feng, Dongxiao Lv, Junfei Wang, Tian Tian, Xingwen Wang

**Affiliations:** 1 Department of Respiratory and Critical Care Medicine, Qilu Hospital of Shandong University, Jinan, Shandong 250012 China; 2 Cancer Center, Shandong Provincial Hospital Affiliated to Shandong First Medical University, Jinan, Shandong 250021 China

**Keywords:** Non-small-cell lung cancer, Cancer metabolism

## Abstract

Growing evidence has highlighted the roles of circular RNAs (circRNAs) in non-small-cell lung cancer (NSCLC), however, their roles in NSCLC glycolysis remains poorly understood. CircRNAs microarray profiles discovered a novel exon-derived circRNA, circSLC25A16 (hsa_circ_0018534), in NSCLC tissue samples. In NSCLC samples, high-expression of circSLC25A16 was associated with unfavorable prognosis. Cellular experiments revealed that circSLC25A16 accelerated the glycolysis and proliferation of NSCLC cells. Besides, circSLC25A16 knockdown repressed the in vivo growth by xenograft assays. RNA-fluorescence in situ hybridization (RNA-FISH) illustrated that circSLC25A16 and miR-488-3p were both located in cytoplasm. Mechanistic experiments demonstrated that circSLC25A16 interacts with miR-488-3p/HIF-1α, which activates lactate dehydrogenase A (LDHA) by facilitating its transcription. Collectively, present research reveals the crucial function of circSLC25A16 on NSCLC glycolysis through miR-488-3p/HIF-1α/LDHA, suggesting the underlying pathogenesis for NSCLC and providing a therapeutic strategy for precise treatment.

## Introduction

Non-small-cell lung cancer (NSCLC) takes the largest proportion of lung cancer, the most common type of cancer, and acts as the leading cause of cancer-related mortality worldwide^1,2^. In early stage, NSCLC is usually asymptomatic, which delays the diagnosis of NSCLC. Recently, the incidence and mortality of NSCLC have been increased and traditional surgical resection is difficult to comprehensively overcome the puzzle^3^. On this basis, the chemotherapy and more accurate molecular targeting therapy are more necessary. In spite of current advances in therapy, the overall five-year survival rate for NSCLC patient still remains poor^4^. Therefore, novel diagnostic approach and therapeutic target are urgently necessary to optimize the prognosis and therapeutic effect.

Circular RNAs (circRNAs) are specific covalent closed circular non-coding RNAs that wildly expressed in eukaryocyte^5,6^. CircRNAs have multiple regulatory functions and mechanisms that modify transcriptional and post-transcriptional regulation^7,8^. For posttranscriptional regulation, circRNAs can act as microRNA (miRNA) sponges or competitively combine with miRNA^9^. CircRNAs play critical roles in various cancers. For instance, circRNA circFGFR1 is upregulated in NSCLC tissues and associated with clinicopathological characteristics and poor prognosis^10^. Circ_0074027 is elevated in NSCLC tissue specimens and cell lines and associated with advanced TNM stages and worse prognosis survival. CircARHGAP10 is observed to be significantly upregulated in NSCLC tissues and cells and its silencing suppresses the proliferation and metastasis via targeting the miR-150-5p/GLUT1 axis. Circ_0074027 directly sponges miR-185-3p to enhance BRD4 and MADD^11^. Overall, these findings suggest the critical roles of circRNAs in NSCLC.

The current investigation is determined to clarify the roles of circSLC25A16 (hsa_circ_0003459) in NSCLC glycolysis and tumor progression. CircSLC25A16 interacts with miR-488-3p and hypoxia-inducible factor 1-alpha (HIF-1α), which activates LDHA by facilitating its transcription. Taken together, this research reveals the molecular mechanisms of circSLC25A16 on NSCLC glycolysis through miR-488-3p/HIF-1α/LDHA, suggesting the underlying pathogenesis for NSCLC and providing a therapeutic strategy for precise treatment.

## Materials and methods

### Tissue samples collection

Forty NSCLC tissue samples and their paired adjacent non-tumor tissues were acquired from patients who underwent the surgical treatment at Qilu Hospital of Shandong University. The tumor samples and paired non-tumor samples were collected in the operation and none of these patients had received chemotherapy or radiotherapy prior to this surgery. Our study was approved by the Ethics Committee of Qilu Hospital of Shandong University and written informed consent was obtained from all these enrolled individuals. Clinicopathological characteristics were summarized in Table 1.

**Table 1:** Clinicopathological feature of NSCLC patients with circSLC25A16 expression.

	Total	circSLC25A16		*p*
		Low = 13	High = 17	
Gender				
Male	18	8	10	0.582
Female	12	5	7	
Age (years)				
≥60	16	7	9	0.542
<60	14	6	8	
TNM				
I–II	10	6	4	0.020*
III/IV	20	7	13	
Lymph metastasis				
No	13	6	7	0.402
Yes	17	7	10	
Differentiation				
Well, moderate	13	8	5	0.187
Poor	17	5	12	

^*^P < 0.05 represents statistical difference.

### Cell lines and culture

Human normal bronchial epithelial cells (NHBE) and NSCLC cell lines (H460, H1299, A549) were purchased from the ATCC cell bank (Manassas, VA, USA). RPMI-1640 medium (Gibco, CA, USA) supplemented with 10% FBS (fetal bovine serum, Gibco) was used to culture the cells in incubator containing 5% CO_2_ atmosphere at 37 °C.

### Transfection

For circRNA silencing, the sh-circSLC25A16 (shRNA directly targeting circRNA) and sh-NC (negative control shRNA) were constructed by GenePharma Biotech (Shanghai, China). Cells were transfected with the recombinant lentiviral transduction particles (GenePharma). The mimics and inhibitor of miR-488-3p and their controls (miR-NC) were provided by RiboBio (Guangzhou, China) (Table S1). After stable transfection, cells were chosen by 1 μg/ml puromycin for two weeks. CircRNA cDNA was amplified and inserted into the overexpression vector (Greenseed Biotech Co, Guangzhou, China) and then transfected using Lipofectamine 2000 (Invitrogen) according to the manufacturer’s instructions.

### Quantitative real-time PCR

Trizol reagent kit (Invitrogen) was used to isolate the total RNA from NSCLC cells or tissues. Then, NanoDrop 2000 spectrophotometer (Thermo Scientific, Wilmington, DE, USA) was used to identify the concentration of RNA. Transcriptor First Strand cDNA Synthesis Kit (Roche, Indianapolis, IN, USA) was used to synthesis cDNA. The expression of circRNA and mRNAs were determined using SYBR Green Real-time PCR Master Mix (Toyobo, Japan) using beta-actin control. The expression of miRNA was determined using miRNA qRT-PCR Starter kit (Riobo) using U6 control. The relative expression was calculated by using 2^−ΔΔCt^ method (Table S1). For RNase R and actinomycin D testing, RNase R (3 U/μg, Epicentre Technologies, Madison, WI, USA) and Act D (5 μg/mL, Sigma, Aldrich, St. Louis, MO, USA) was administrated to NSCLC cells. Then, cells were collected and the expression of circSLC25A16 and mRNA were analyzed using RT-qPCR as described above.

### Glucose consumption and lactate production

The glucose consumption, lactate production and ATP analysis were measured as previous described^12^. For glucose uptake assay, the glucose consumption was performed using colorimetric glucose assay kit (BioVision, Milpitas, CA, USA) and normalized according to cell number. For lactate assay, the lactate production was detected using lactate assay kit (BioVision) according to the manufacturer’s instructions. For ATP analysis, the ATP levels were analyzed using ATP assay kit (Beyotime) according to the manufacturer’s instructions.

### Extracellular acidification rate (ECAR)

ECAR was determined using the Seahorse XFe96 analyzer (Seahorse Bioscience, Agilent). Briefly, the transfected A549 cells (1 × 10^4^ cells/well) were seeded in 96-well XF cell culture microplates and cultured in medium. After 24 h, cells were cultured in XF base medium (pH 7.4) added with glucose (10 mM), glutamine (1 mM), 2-DG (50 mM) and oligomycin (1 µM). Finally, ECAR was measured using XF96 analyzer (Seahorse Bioscience). The data was analyzed by Seahorse XF Glycolysis Stress Test Report Generator package.

### Ethynyl-2-deoxyuridine (EdU) incorporation and cell counting Kit-8 (CCK-8) assay

EDU incorporation assay was carried out to evaluate NSCLC cells’ proliferation ability using 5-ethynyl-2-deoxyuridine (EdU) labeling/detection kit (Ribobio, Guangzhou, China) according to the manufacturer’s protocol. For CCK-8 assays, A549 cells (2 × 10^3^) were seeded in 96-well plates supplemented with growth medium. At 1, 2, 3, 4 days after transfection, CCK-8 solution (10 μl) was added to each well for incubation. Then, the absorbance was measured at an optical density (450 nm) using Microplate reader (Bio-Rad, Hercules, CA).

### Western blot analysis

NSCLC tissue or cellular proteins were respectively extracted with cell lysis buffer (Promega, Madison, WI, USA). Thirty micrograms of proteinextraction was separated on an 8% SDS-PAGE gel and then transferred to nitrocellulose membranes. After blockage by milk, the membranes were incubated for 2 h at room temperature with primary antibody (anti-HIF1A, 1:1000 dilution, Abcam, ab51608; anti-LDHA, ab101562, 1:1000 dilution, Abcam). Then, blots were incubated at room temperature for 90 min with horseradish peroxidase (HRP) conjugated beta-actin secondary antibodies (diluted 1:5000). The bands were scanned and visualized by GS700 imaging densitometer (Bio-Rad Laboratories) and analyzed by Image Studio software.

### RNA-fluorescence in situ hybridization (RNA-FISH)

Cy3-labeled probe sequences for circSLC25A16 and FAM-labeled probe for miR-488-3p were constructed by Genepharma (Shanghai, China). RNA FISH were performed for analysis of the co-localization of circSLC25A16 and miR-488-3p in NSCLC cells using fluorescent in situ hybridization kit (Genepharma) according to the manufacturer’s protocol. The detailed description was reported as previous^13^. The cell nucleus were counterstained with 4,6-diamidino-2-phenylindole (DAPI). The image of FISH was obtained under confocal microscope (Olympus).

### Subcellular fractionation location

For subcellular fractionation location, PARIS Kit (Life Technologies, CA, USA) was performed to separate nuclear and cytosolic fractions from NSCLC cells according to the manufacturer’s instructions. U1 and GAPDH acted as the expression standard in cytoplasm and nuclear fraction.

### Luciferase reporter assay

The interactions within circSLC25A16, miR-488-3p, HIF-1α, and LDHA were determine luciferase reporter assay. In brief, vectors were constructed using psicheck2-based plasmid (Promega, Madison, WI, USA) containing circSLC25A1 or LDHA mRNA 3’-UTR (named as psicheck2-circSLC25A16 or psicheck2-control, psicheck2-LDHA or controls). Besides, mutants were also constructed. 293T cells were co-transfected with the psicheck2-based plasmids with miR-488-3p mimics or inhibitors (GenePharma, Shanghai, China). After 24 h transfection, the luciferase activity was measured using Renilla Luciferase Assay System (Promega, Madison, WI, USA) according Firefly and Renilla luciferase activity.

### Chromatin immunoprecipitation (ChIP)-PCR

The HIF-1α-binding sites surrounding LDHA promoter region were predicted using an in silico analysis tools (JASPAR, http://jaspar.genereg.net/). In brief, 1 × 10^7^ transfected cells were sonicated and crosslinked chromatin was decomposed to be 200–1000 bp fragments. Anti-HIF-1 alpha antibody (Abcam, ab51608) were used to precipitate protein-DNA complexes. Normal immunoglobulin G (IgG) acted as a negative control. The enrichment of precipitation was detected by quantitative real-time PCR using 2^−ΔΔCt^ method. IgG was used as a negative control.

### Tumor xenografts

Approximately 2 × 10^6^ A549 cells resuspended in 100 μl PBS were subcutaneously injected into the flank of BALB/c nude mice (total 10 male mice, five-week old). Mice were sacrificed after four weeks later by chloral hydrate injection. The neoplasm was orderly removed and weighed. Tumor volume of xenografts was calculated using *a* × *b*^2^/2 (a for the long diameter and b for short diameter). This animal study was approved by the Ethics Committee of Qilu Hospital of Shandong University.

### Statistical analysis

Data was presented as mean ± standard deviation (SD). Paired/non-paired two-tailed t-test or one-way ANOVA analysis was conducted for the groups’ comparisons. Linear regression and Pearson-correlation coefficient were performed to evaluate the correlation between circRNA and mRNA expression. *p*-value < 0.05 was considered significant.

## Results

### CircSLC25A16 was upregulated in the NSCLC

**Fig. 1:**
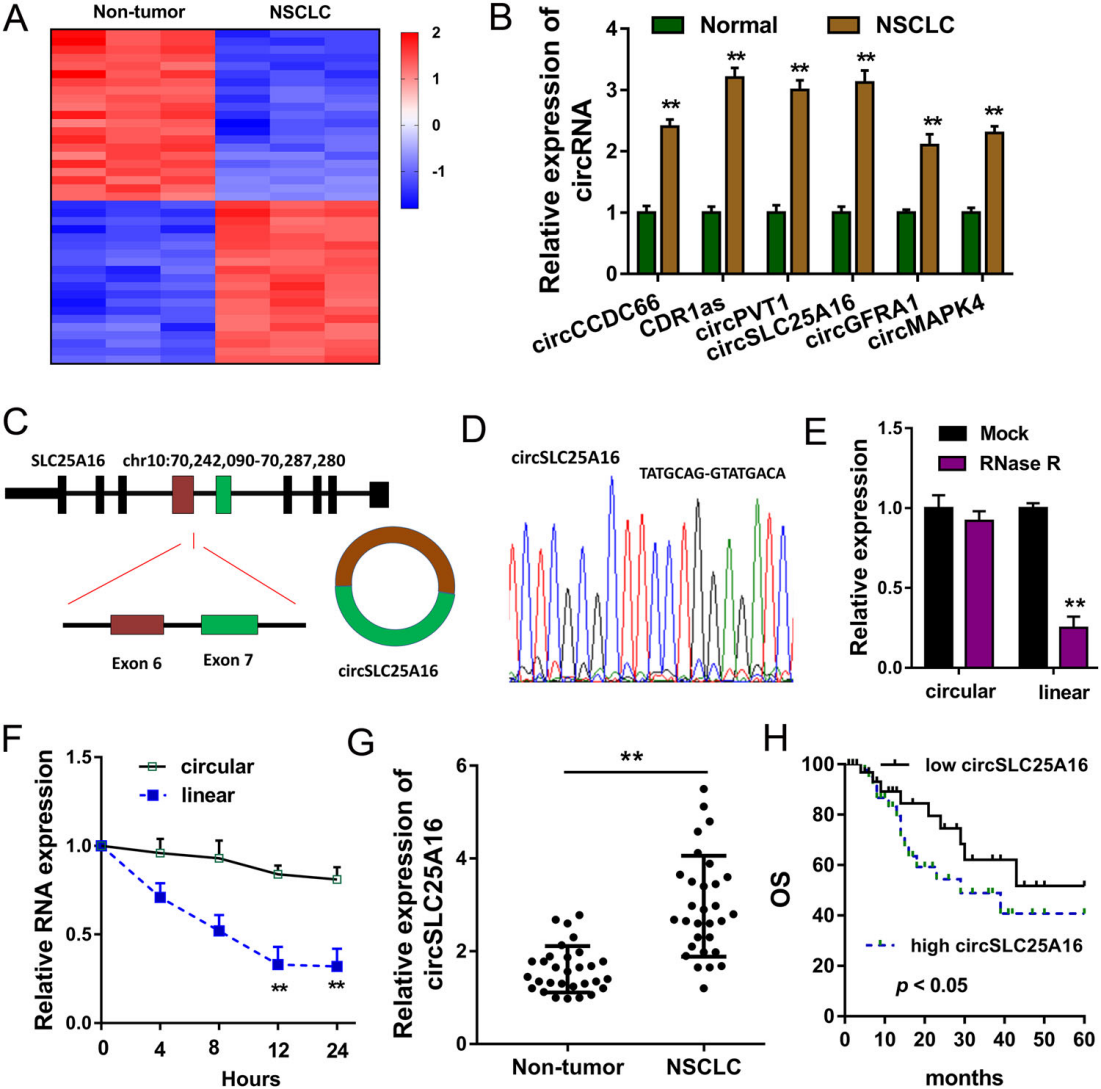
CircSLC25A16 was upregulated in the NSCLC. **a** Heatmap revealed the series of dysregulated circRNAs using circRNA microarray in the NSCLC tissue samples and non-tumor normal tissue. **b** RT-PCR revealed the expression of upregulated circRNAs, including circCCDC66, CDR1as, circPVT1, circSLC25A16, circGFRA1, and circMAPK4. **c** Specification for the generation of circSLC25A16 from SLC25A16 gene exon 7 and exon 6 by back-splicing. **d** Sanger sequencing confirmed the conjunction site of circSLC25A16 and covalent closed loop. **e** RT-PCR revealed the expression of SLC25A16 mRNA or circSLC25A16 after RNase R administration. **f** RT-PCR revealed the levels SLC25A16 mRNA and circSLC25A16 with Actinomycin D administration. **g** RT-PCR illustrated the circSLC25A16 presentation in NSCLC tissue comparing to normal tissue. **h** The unfavorable prognosis of NSCLC tissue with high level of circSLC25A16. Data are presented as the mean ± SD. **p < 0.01 versus the control group.

Using the circRNA microarray, our team found that a series of circRNAs were dysregulated in the NSCLC tissue samples and non-tumor normal tissue (Fig. 1a). Moreover, these circRNAs were validated to be upregulated in NSCLC, including circCCDC66, CDR1as, circPVT1, circSLC25A16, circGFRA1 and circMAPK4 (Fig. 1b). CircSLC25A16 was a novel circRNA generated from SLC25A16 gene exon 7 and exon 6 by back-splicing (Fig. 1c). Sanger sequencing found that the conjunction site of circSLC25A16 and confirmed the covalent closed loop (Fig. 1d). RNase R administration showed that the linear SLC25A16 mRNA was digested and the circular forms of circSLC25A16 was stable (Fig. 1e). Actinomycin D administration showed that circSLC25A16 was stable more than linear SLC25A16 mRNA (Fig. 1f). In the NSCLC tissue, circSLC25A16 was upregulated comparing to normal tissue (Fig. 1g). More than that, the overexpression of circSLC25A16 indicated the unfavorable prognosis of NSCLC tissue (Fig. 1h). Thus, circSLC25A16 was upregulated in the NSCLC.

### CircSLC25A16 accelerated the glycolysis of NSCLC

**Fig. 2:**
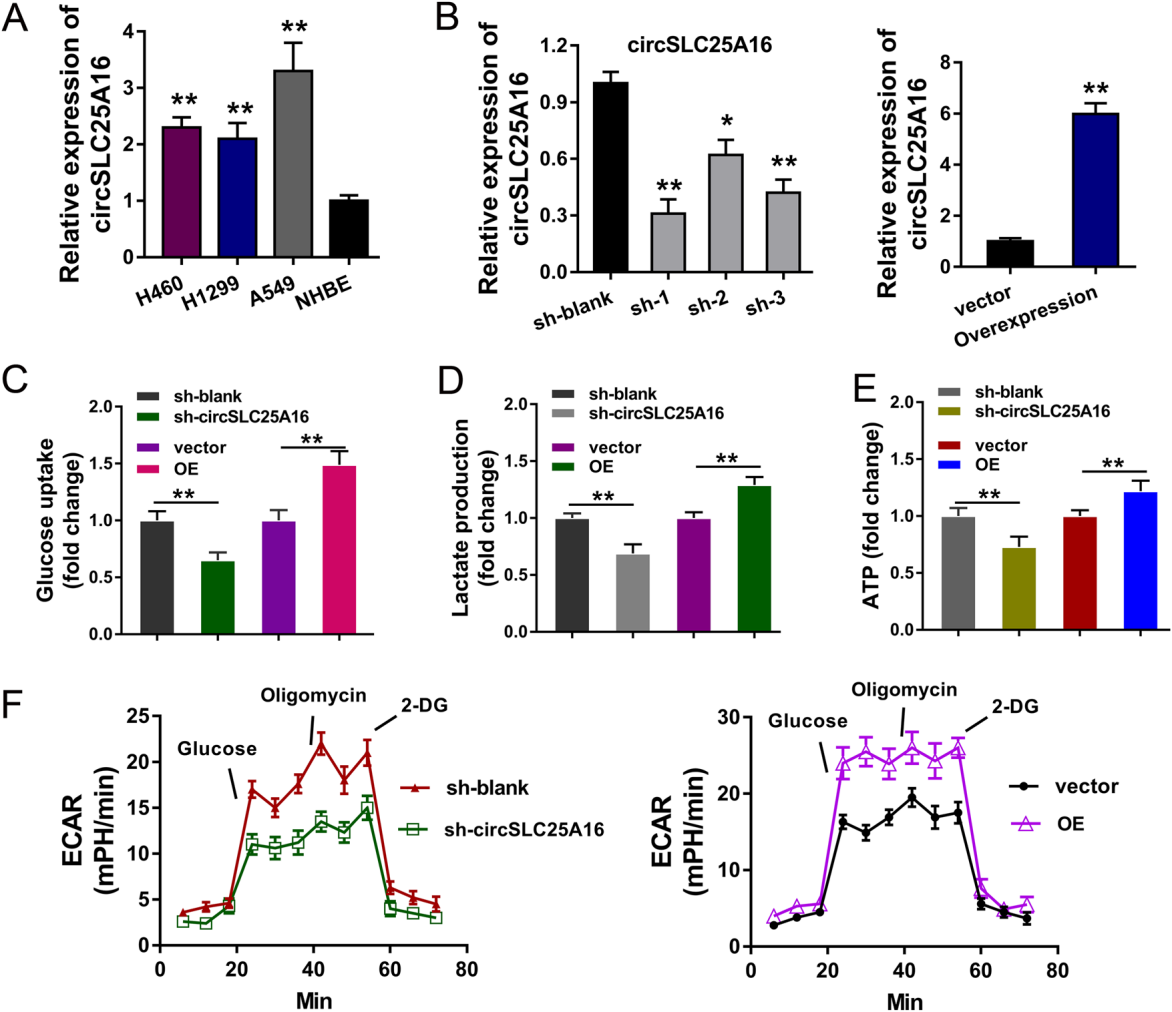
CircSLC25A16 accelerated the glycolysis of NSCLC. **a** The expression of circSLC25A16 was detected using RT-PCR in NSCLC cells (H460, H1299, A549) and normal cells (NHBE). **b** In A549 cells, shRNAs specifically targeting circSLC25A16 were transfected to knock down its expression, and overexpression plasmids (OE) was transfected to enhance circSLC25A16 expression. **c** Glucose uptake analysis, **d** lactate production analysis and **e** ATP analysis demonstrated the glucose utilization in A549 cells transfected with circSLC25A16 shRNA (sh-circSLC25A16) and circSLC25A16 overexpression (OE), as well as controls. **f** ECAR analysis (extracellular acidification rate) showed the glycolytic capacity of A549 cells transfected with circSLC25A16 shRNA (sh-circSLC25A16) and circSLC25A16 overexpression (OE). *p < 0.05. **p < 0.01.

In the NSCLC cells (H460, H1299, A549), the expression of circSLC25A16 was detected using RT-PCR, illustrating the high-expression of circSLC25A16 (Fig. 2a). For the functional experiments, shRNAs specifically targeting circSLC25A16 were transfected into A549 cells to knock down its expression, and overexpression plasmids (OE) was transfected to enhance circSLC25A16 expression (Fig. 2b). The glucose uptake analysis demonstrated that knockdown of circSLC25A16 suppressed the glucose utilization, and circSLC25A16 overexpression promoted the glucose intake (Fig. 2c). The lactate production analysis demonstrated that knockdown of circSLC25A16 suppressed the lactate accumulation, and circSLC25A16 overexpression promoted the accumulation (Fig. 2d). The ATP analysis demonstrated that knockdown of circSLC25A16 suppressed the ATP level, and circSLC25A16 overexpression promoted it (Fig. 2e). Moreover, ECAR analysis found that knockdown of circSLC25A16 suppressed the glycolytic capacity, and circSLC25A16 overexpression promoted it (Fig. 2f). Taken together, these data above suggest that circSLC25A16 accelerated the glycolysis of NSCLC.

### CircSLC25A16 regulated the proliferation of NSCLC

**Fig. 3:**
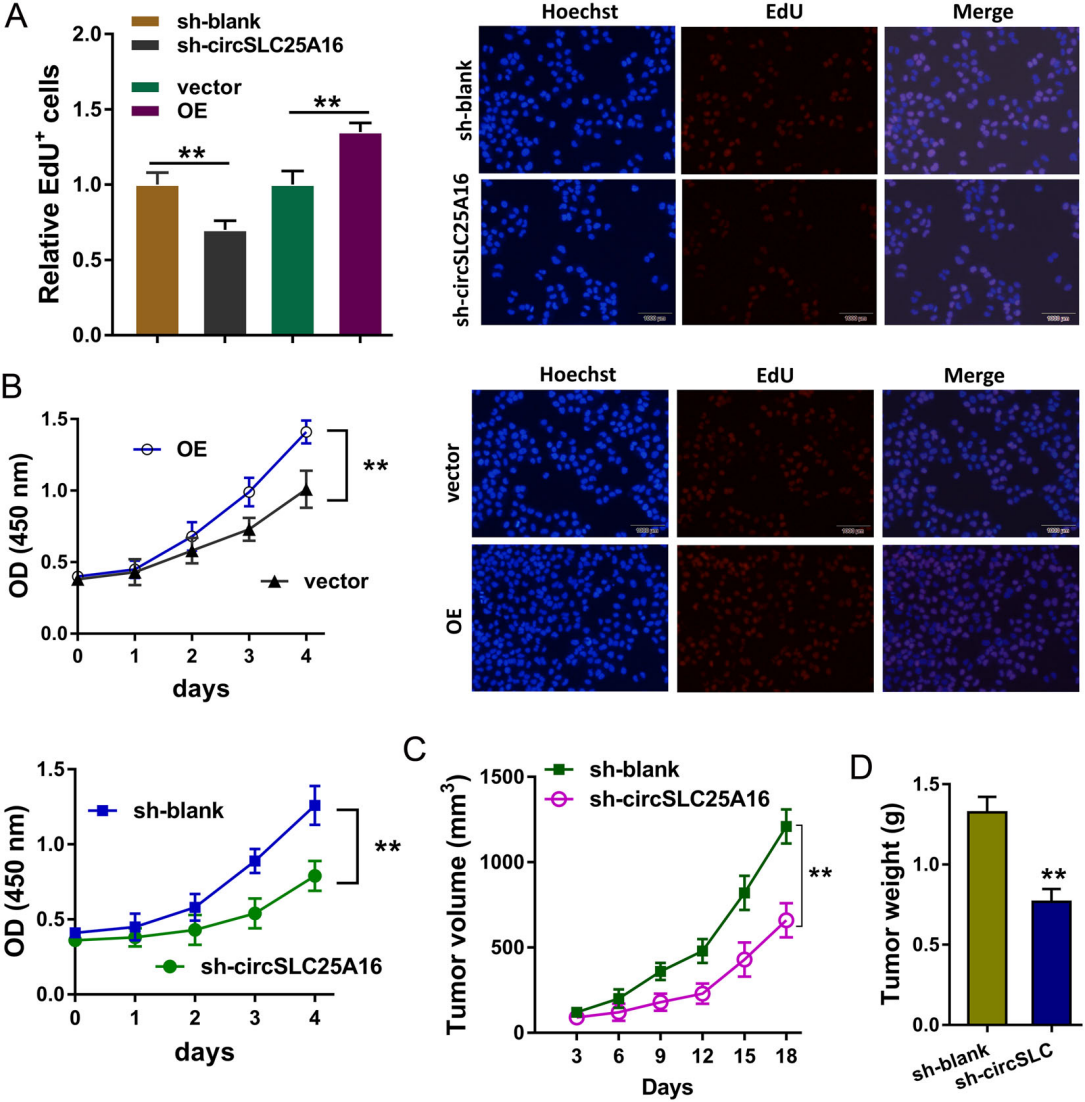
CircSLC25A16 regulated the proliferation of NSCLC. **a** EdU analysis showed the EdU positive A549 cells transfected with circSLC25A16 knockdown, circSLC25A16 overexpression (OE) and controls. **b** CCK-8 analysis showed the EdU positive A549 cells transfected with circSLC25A16 knockdown, circSLC25A16 overexpression (OE) and controls. **c** In vivo mice xenograft assay illustrated the tumor volume and **d** neoplasm weight using the A549 cells transfected with sh-circSLC25A16. **p < 0.01.

In order to investigate the roles of circSLC25A16 in NSCLC cells’ proliferation, CCK-8 assay and EdU analysis were performed. EdU analysis showed that circSLC25A16 knockdown suppressed the EdU positive cells, and circSLC25A16 overexpression promoted EdU positive cells (Fig. 3a). CCK-8 assay showed that circSLC25A16 knockdown suppressed the proliferation, and circSLC25A16 overexpression promoted it (Fig. 3b). In vivo mice xenograft assay illustrated that circSLC25A16 knockdown suppressed the tumor volume (Fig. 3c) and neoplasm weight (Fig. 3d) using the A549 cells transfected with sh-circSLC25A16. Taken together, these data above suggest that CircSLC25A16 regulated the proliferation of NSCLC.

### miR-488-3p acted as the target of circSLC25A16

**Fig. 4:**
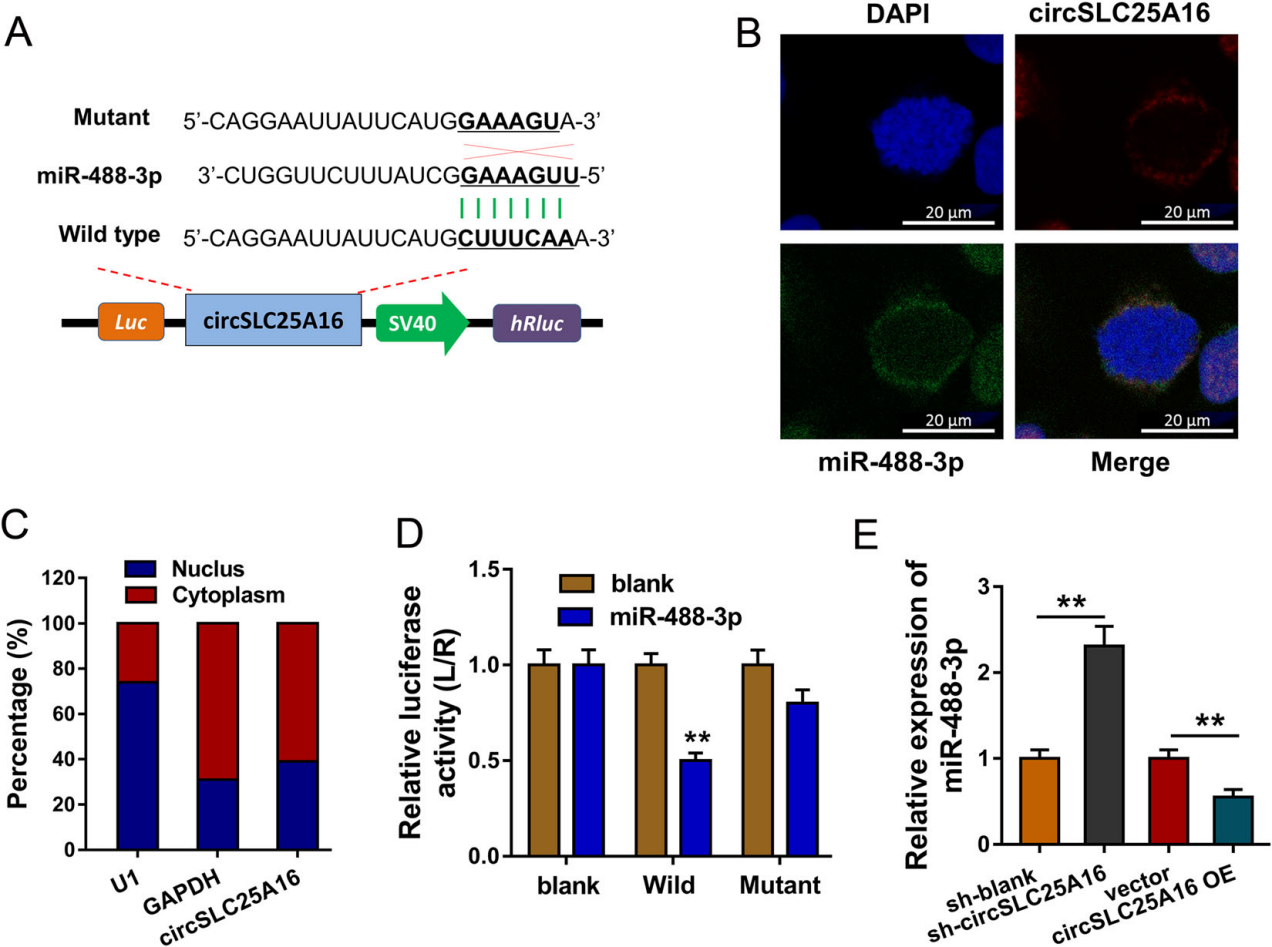
miR-488-3p acted as the target of circSLC25A16. **a** Investigation for the potential miRNAs of circSLC25A16 reveals that circSLC25A16 targets miR-488-3p as a classic miRNA sponge. Wild type and mutant sequences were constructed for the luciferase reporter assay. **b** RNA-FISH analysis indicated the cytoplasmic segment or nucleus segment of circSLC25A16 in A549 cells. **c** Subcellular location analysis by RT-PCR found the location of circSLC25A16 in cytoplasm or nucleus. **d** Luciferase reporter assay presented the luciferase activity with the miR-488-3p mimics or control and circSLC25A16 mutant or wild type. **e** RT-PCR indicated the miR-488-3p expression in A549 cells using the circSLC25A16 knockdown and overexpression transfection. **p < 0.01.

To investigate the potential regulation of circSLC25A16, we found that circSLC25A16 could target miRNAs as a classic miRNA sponge, especially miR-488-3p (Fig. 4a). Then, we investigate the subcellular location of circSLC25A16 in A549 cells. RNA-FISH analysis indicated that circSLC25A16 was distributed in the cytoplasmic segment (Fig. 4b). Subcellular location analysis found that circSLC25A16 was more located in the cytoplasm than the nucleus (Fig. 4c). For the luciferase reporter assay, the wild type and mutant sequences were constructed and the luciferase activity analysis found that circSLC25A16 closely combined with the miR-488-3p through covalent molecular conjunction (Fig. 4d). RT-PCR indicated that, using the circSLC25A16 knockdown and overexpression transfection, miR-488-3p expression was significantly upregulated or reduced (Fig. 4e). Taken together, these data above suggest that miR-488-3p acted as the target of circSLC25A16.

### HIF-1α served as the target of circSLC25A16/miR-488-3p

**Fig. 5:**
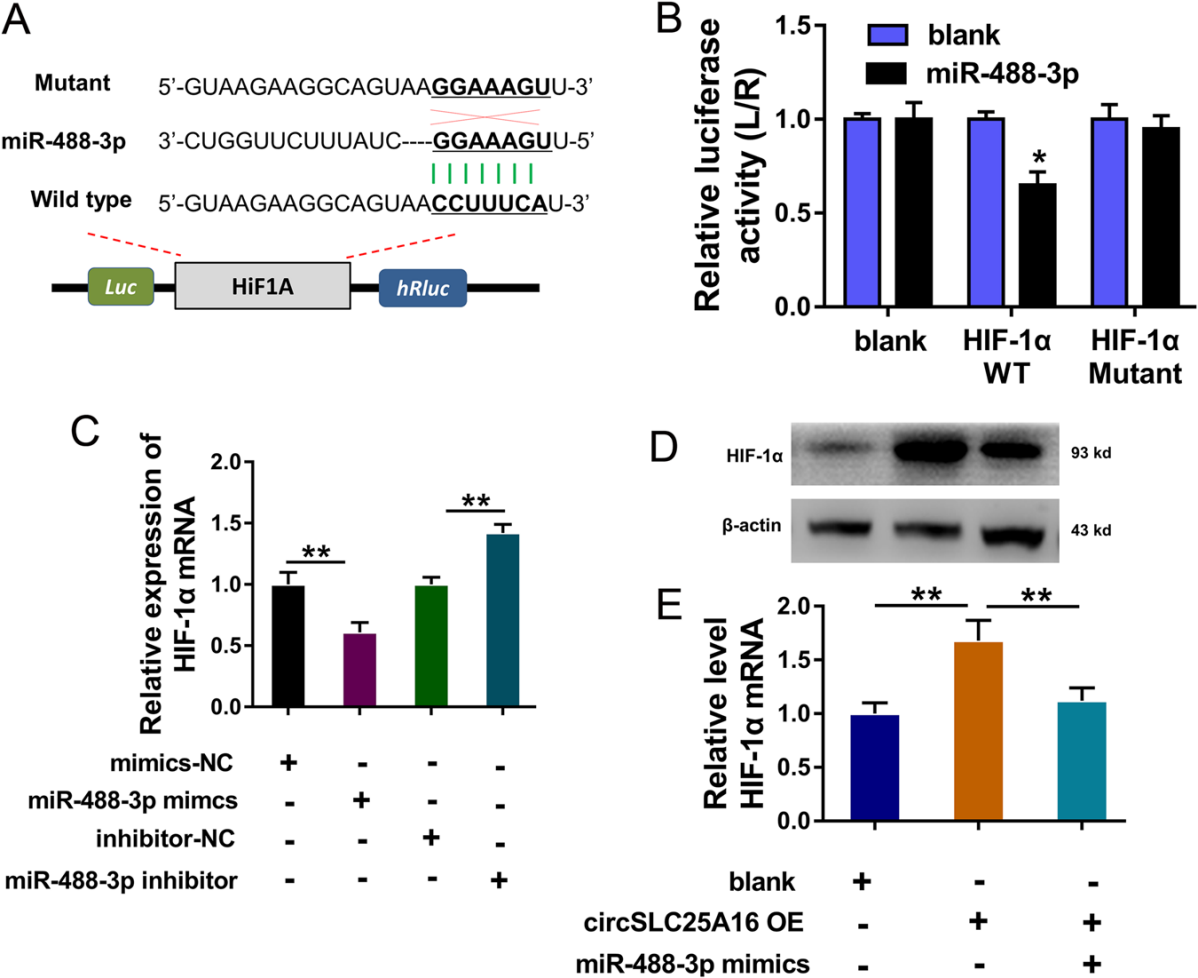
HIF-1α served as the target of circSLC25A16/miR-488-3p. **a** Bioinformatics prediction revealed the potential target effector protein for miR-488-3p. The wild type and mutant sequences for HIF-1αluciferase reporter vector were constructed. **b** Luciferase reporter assay demonstrated the activities of miR-488-3p mimics/control combined with the HIF-1α mRNA wild type/mutant. **c** RT-PCR revealed the HIF-1α mRNA expression in A549 cells transfected with miR-488-3p mimics or inhibitor. **d** Western blot and **e** RT-PCR revealed the HIF-1α protein and mRNA with circSLC25A16 overexpression (circSLC25A16 OE) transfection and miR-488-3p mimics co-transfection. **p < 0.01. *p < 0.05.

Due to the finding that miR-488-3p acted as the target of circSLC25A16, we further investigate the potential target for the circRNA/miRNA axis. Bioinformatics prediction revealed that HIF-1α might function as one target effector protein for circSLC25A16/miR-488-3p (Fig. 5a). For the luciferase reporter assay, the wild type and mutant sequences were constructed and the luciferase activity analysis found that miR-488-3p mimics closely combined with the HIF-1α mRNA wild type sites through covalent molecular conjunction (Fig. 5b). RT-PCR revealed that, using miR-488-3p mimics or inhibitor transfection, HIF-1α mRNA expression was significantly decreased or upregulated (Fig. 5c). Western blot and RT-PCR revealed that circSLC25A16 overexpression (circSLC25A16 OE) transfection enhanced the HIF-1α protein (Fig. 5d) and mRNA (Fig. 5e), and the miR-488-3p mimics co-transfection rescued the protein or mRNA. Taken together, these data above suggest that HIF-1α served as the target of circSLC25A16/miR-488-3p.

### HIF-1α facilitated the transcriptional level of LDHA

**Fig. 6:**
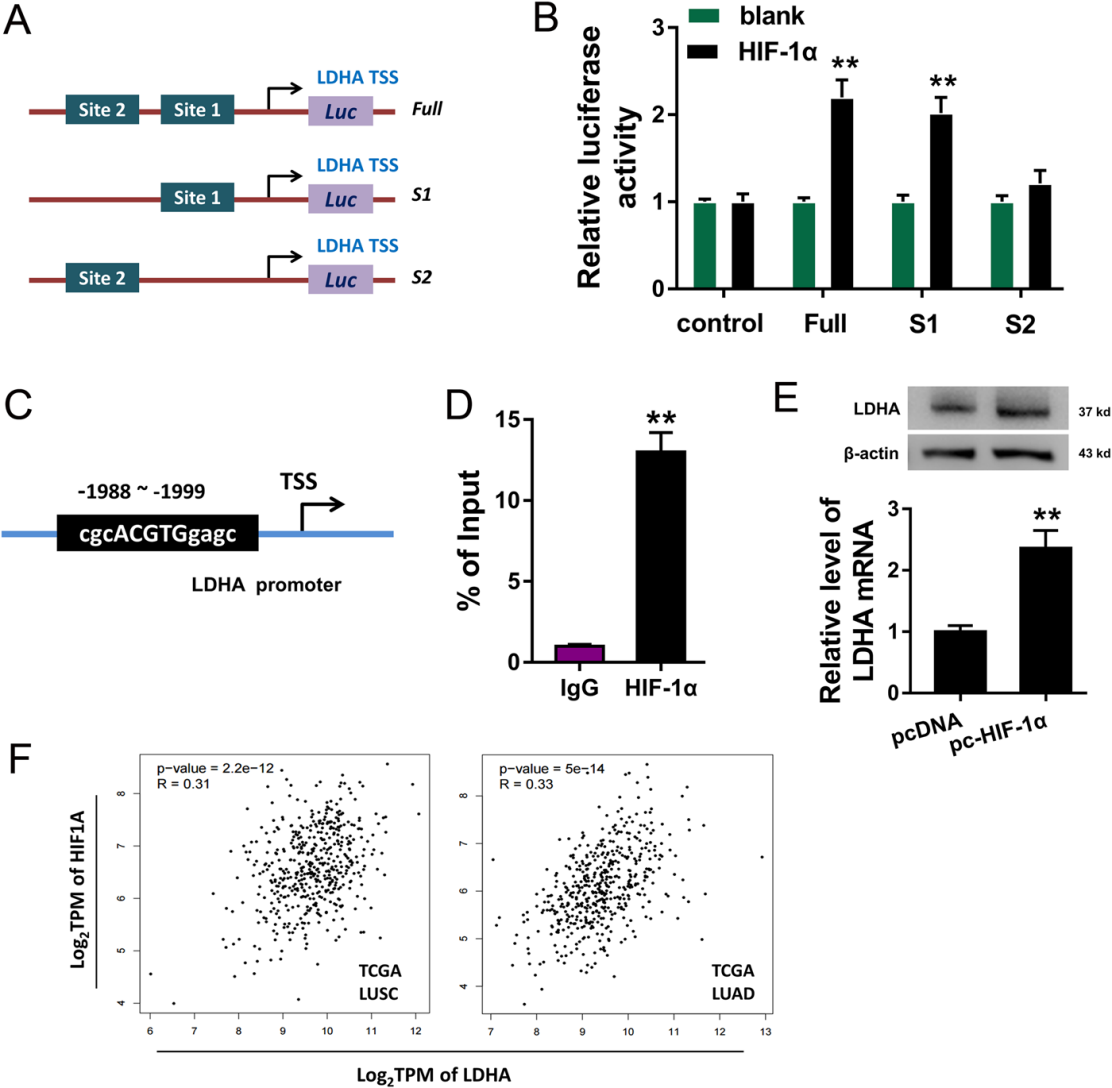
HIF-1α facilitated the transcriptional level of LDHA. **a** Bioinformatics tools (JASPAR, http://jaspar.genereg.net/) demonstrated the potential binding sites in the promoter region of LDHA. **b** Luciferase reporter vectors were constructed for the two binding sites. Relative luciferase activities were detected. **c** For the chromatin immunoprecipitation (ChIP) assay, the primers were synthesized based on the binding sites. **d** ChIP-qPCR showed the enrichment of LDHA promoter region using primers. **e** Western blotting and RT-PCR showed the mRNA and protein level of LDHA transfected with HIF-1α overexpression (pc-HIF-1α) or control. **f** The public database (TCGA, http://gepia.cancer-pku.cn/index) demonstrated the correlation within LDHA expression and HIF-1α expression in LUSC (lung squamous carcinoma) and LUAD (lung adenocarcinoma) cohort. **p < 0.01.

According to this finding that HIF-1α functioned as the effector of circSLC25A16/miR-488-3p, we further explored the potential role of the transcription factor HIF-1α. Bioinformatics tools (JASPAR, http://jaspar.genereg.net/) found that there were two potential binding sites in the promoter region of LDHA (Fig. 6a). Luciferase reporter vectors were constructed for the two binding sites and results revealed that HIF-1α could effectively bind with the first binding sites of LDHA promoter region (Fig. 6b). For the ChIP assay, the primers were synthesized based on the binding sites and the abundance in precipitation was detected (Fig. 6c). ChIP-qPCR showed that transcriptional factor HIF-1α significantly connect with the binding sites of LDHA promoter region (Fig. 6d). Western blotting and RT-PCR showed that HIF-1α overexpression (pc-HIF-1α) could enhance the mRNA and protein level of LDHA (Fig. 6e). The public database (TCGA, http://gepia.cancer-pku.cn/index) demonstrated that HIF-1α expression was positively correlated with LDHA expression in LUSC (lung squamous carcinoma) and LUAD (lung adenocarcinoma) cohort (Fig. 6f). Taken together, these data above suggest that HIF-1α facilitated the transcriptional level of LDHA.

## Discussion

The crucial regulations of circRNAs on human lung cancer have been incrementally recognized by more and more researchers. In multiple levels of NSCLC, circRNAs modulate the differentiation, proliferation, migration and metastasis through series of regulative patterns, including competing endogenous RNA, transcriptional silencing complex, histone modification and so on. Distinctively, at multiple regulation of NSCLC tumorigenesis, circRNAs have been confirmed to regulate the epithelial mesenchymal transformation^14^, invasion and migration^15^. Based on these new findings, we could be aware of the crucial functions of circRNAs in NSCLC.

As regarding to one of the canonical tumor cell traits, glycolysis (also known as Warburg effect) is considered as a critical phenotype. However, the role and molecular mechanism by which circRNAs regulate NSCLC glycolysis are still unclear. In present research, we found that the newfound circRNA, named as circ circSLC25A16, was significantly upregulated in the NSCLC tissue and cells. Moreover, the remarkable overexpression of circSLC25A16 was correlated with the unfavorable prognosis of NSCLC patients. In the clinical analysis, we found that the stage III-IV NSCLC group had a higher circSLC25A16 expression. This finding illustrated that circSLC25A16 might be relevant to the unfavorable tumor grading. Functional assay found that circSLC25A16 promoted the glycolysis phenotype of NSCLC cells, including glucose uptake, lactate production and ATP yielding capacity, as well as the ECAR. As regarding to tumor phenotype, functional assay found that circSLC25A16 promoted the proliferation of NSCLC cells. Using these evidence, we found that circSLC25A16 may function as an oncogene in the NSCLC tumorigenesis by promoting the glycolysis and proliferation.

Glycolysis is an important feature for tumor cells, which provides the main source of energy for its rapid expansion growth. Abundant evidence has demonstrated that circRNAs could regulate the glycolysis of cancer cells. For example, a novel circRNA circMYC promotes the proliferation and glycolysis of human melanoma cells by directly binding to miR-1236, as a miRNA sponge, then targets LDHA to accelerate the glycolysis^16^. CircRNA ciRS-122 (hsa_circ_0005963) is positively correlated with chemoresistance of colorectal cancer, and is determined to acts as miR-122 sponge for targeting PKM2, which was delivered by the exosomes from oxaliplatin-resistant cells^17^. A ZNF292-spliced circRNA, has-circRNA-403658, is upregulated in hypoxia-induced bladder cancer cells. Has-circRNA-403658 silencing inhibits the LDHA-mediated aerobic glycolysis, thereby the growth and proliferation of bladder cancer cells^18^.

Up to now, most identified circRNA were derived from the exon of human genes. In contrast, only a fraction of circRNAs are generated from introns^19^. An interesting tendency is found for the interaction within circRNA generation and their functions^20,21^. The general phenomenon is that the exon-generated circRNAs are mostly located in the cytoplasm and function as miRNA sponge, and the intron-generated circRNAs are mostly located in the nuclear and function as covalent binding element^22^. Here, our research found that circSLC25A16 performs as a sponge for miR-488-3p and miR-488-3p targets the 3′-UTR of HIF-1α mRNA, thereby constructing the axis of circSLC25A16/miR-488-3p/HIF-1α. Because the target effector of circSLC25A16 is HIF-1α, we could consider that circSLC25A16 exerts the regulation for glycolysis through regulating HIF-1α. Moreover, HIF-1α is a hypoxia related transcriptional factor and we found that HIF-1α could inspire the transcription level of LDHA mRNA. Besides, this finding of HIF-1α/LDHA axis has also been identified in human cancer^23^.

In summary, this work demonstrated that circSLC25A16 is upregulated in NSCLC tissues/cells, and associated with unfavorable outcome of NSCLC patients. CircSLC25A16 interacts with miR-488-3p/HIF-1α and activates LDHA by facilitating its transcription. Hence, the key molecular mechanisms of circRNA and glycolysis underlying NSCLC occurrence and progression are critical for precise treatment.

## Supplementary information

Table S1
