# Supplementary material for: Circular RNA circSLC25A16 contributes to the glycolysis of non-small-cell lung cancer through epigenetic modification
Source: Cell Death Dis. 2020 Jun 8;11(6):437. doi: 10.1038/s41419-020-2635-5 (PMC7280231; doi:10.1038/s41419-020-2635-5)
Supplement: Supplementary file 1 — Table S1 [file 41419_2020_2635_MOESM1_ESM.docx]

**Supplement Table 1**. Sequences of shRNA and qRT-PCR primers.

|  | 5’-3’ |
| --- | --- |
| circSLC25A16 | forward, 5’-ATGCAAAGGAAGGTGGTTTCT-3'  reverse, 5'-CCCCTTTCACCTGGAATGCT-3’ |
| sh-circSLC25A16-1 | 5’-ATGCAGGTATGACAGCAGTTA-3’ |
| sh-circSLC25A16-2 | 5’-GCTCCATATGCAGGTATGACA-3’ |
| sh-circSLC25A16-3 | 5’-TGGCTCCATATGCAGGTATGA-3’ |
| miR-488-3p | Provided by RiboBio Biotec |
| HIF-1a | forward, 5’-GAACGTCGAAAAGAAAAGTCTCG-3’  reverse, 5’-CCTTATCAAGATGCGAACTCACA-3’ |
| LDHA | forward, 5’-ATGGCAACTCTAAAGGATCAGC-3’  reverse, 5’-CCAACCCCAACAACTGTAATCT-3’ |
| U6 | forward, 5’-CTCGCTTCGGCAGCACA-3’  reverse, 5’-AACGCTTCACGAATTTGCGT-3’ |
| beta-actin | forward, 5’-GGAGCGAGATCCCTCCAAAAT-3’  reverse, 5’-GGCTGTTGTCATACTTCTCATGG-3’ |
| FISH probe | 5'-ACAGCTCGAACACGAAGATGAAAAACTCACTGT  TGGTGACAGATTCTGCAAACGCTGGGG-3’ |
